# Overexpression of cytoplasmic β-catenin inhibits the metastasis of the murine osteosarcoma cell line LM8

**DOI:** 10.1186/1475-2867-14-31

**Published:** 2014-04-02

**Authors:** Teruki Kidani, Atsushi Nakamura, Setsuya Kamei, Yoshiaki Norimatsu, Hiromasa Miura, Hiroshi Masuno

**Affiliations:** 1 Department of Bone and Joint Surgery, Ehime University Graduate School of Medicine, Toon, Ehime 791-0295, Japan; 2 Department of Medical Technology, Faculty of Health Sciences, Ehime Prefectural University of Health Sciences, Takooda, Tobe-cho, Iyo-gun, Ehime 791-2101, Japan

**Keywords:** LM8 murine osteosarcoma cells, Metastatic potential, β-Catenin, Genistein, Matrix metalloproteinase-2

## Abstract

**Background:**

Previously, we found that treatment of LM8 murine osteosarcoma cells with genistein, an isoflavone found in soy, increased the cellular level of β-catenin and decreased its invasive and motile potential. The purpose of this study is to investigate whether the expression of β-catenin in LM8 cells is associated with metastatic potential in nude mice. To this end, we used untreated and genistein-treated LM8 cells.

**Methods:**

LM8 cells were treated for 3 days with or without 50 μM genistein and harvested by trypsinization. Untreated (the control group) and genistein-treated (the genistein group) cells were subcutaneously inoculated into the backs of male nude mice. After 25 days of inoculation, the tumors, lungs, and livers were excised, fixed in 10% formalin, and embedded in paraffin. The sections of formalin-fixed, paraffin-embedded lungs and livers were stained with hematoxylin-eosin (H&E) to confirm the absence or presence of metastatic tumors. The expression of β-catenin within the primary tumor was immunohistochemically examined.

**Results:**

All mice in the control group (n = 8) exhibited large primary tumors, while in the genistein group (n = 8), one mouse showed no tumor formation and the remaining seven mice exhibited smaller primary tumors compared with the control group. The tumor mass of the genistein group was 23% of that of the control group. In the control group, multiple metastatic tumors were found in the lung and/or liver and the metastatic incidence was 100% in the lung and 87.5% in the liver. Six of seven tumor-bearing mice in the genistein group developed no metastatic tumors in the lung or liver, and this group was termed the genistein/metastasis(-) subgroup. Positive β-catenin immunostaining was observed in the cytoplasm of tumor cells, and the β-catenin-labeling index was higher in the genistein/metastasis(-) subgroup than in the control group. The intensity of cytoplasmic β-catenin immunostaining was stronger in the genistein/metastasis(-) subgroup compared with the control group, and the β-catenin-labeling score was 1.9-times higher in the former subgroup than in the latter group.

**Conclusions:**

Overexpression of cytoplasmic β-catenin in LM8 cells causes inhibition of the growth of primary tumors and loss of the metastatic potential to the lung and liver.

## Background

Osteosarcoma is the most common malignant musculoskeletal tumor and occurs mainly in the metaphyseal region of long bones in young people [1,2]. Osteosarcoma expands into the cortex of the bone, later erupts through the cortex into the soft tissues, and often leads to the development of micrometastases in the lung prior to diagnosis. The primary treatment of osteosarcoma is the complete removal of tumor by wide excision with neo-adjuvant and adjuvant chemotherapy [3]. Recently, Spina et al. [4] reported that combination chemotherapy with conventional chemotherapeutic drugs and compounds that increase the therapeutic index of the drug may be useful for the treatment of osteosarcoma. Despite progress in chemotherapy, however, the development of metastatic tumors in the lung often has a fatal outcome [2,5,6]. Therefore, the determination of a possible diagnostic marker for metastatic potential of primary tumor cells is critical for the improvement of prognosis in patients with osteosarcoma.

The initial step of metastasis is cell detachment from the primary tumor. It is well known that mutual adhesiveness of tumor cells is decreased compared with the corresponding normal cells [7]. Cell-cell adhesion molecules, such as catenins and cadherins, play a pivotal role in the maintenance of cell-cell adhesion and normal tissue architecture. β-Catenin is a cytoplasmic molecule, interacts with the cytoplasmic domain of cadherins, and supports the adhesion capability of cadherins [7]. Previously, we identified the loss of membranous β-catenin in LM8 murine osteosarcoma cells [8], which possess extremely high metastatic potential to the lung [9-12]. Hugh et al. [13] reported that loss of membranous β-catenin occurred commonly in primary colorectal cancers with metastatic potential and in the corresponding colorectal liver metastases. Thus, loss of β-catenin at the cell surface seems to be associated with tumor metastasis. However, the association of the level of cytoplasmic β-catenin with the metastatic potential of tumor cells remains unclear.

Genistein is an isoflavone found in dried and green soybeans and soy products, such as soy sauce, miso (fermented soybean paste), and tofu (soybean curd). Experimental studies have shown that genistein inhibits the growth, invasion, and metastasis of tumors *in vivo* and *in vitro*[8,14-16]. Previously, we found that treatment of LM8 cells with genistein inhibited cell proliferation, decreased the expression and secretion of matrix metalloproteinase 2 (MMP-2), which plays a pivotal role in tumor growth, invasion and metastasis [17-19], and decreased cell invasive and motile potential [8]. Moreover, this treatment induced morphological changes, markedly decreased the formation of multilayer masses, and increased the level of osteocalcin mRNA [8]. Thus, genistein may induce the differentiation of LM8 cells. These findings raise the question of whether genistein-treated LM8 cells have the potential to metastasize to the lung *in vivo*.

To explore the above question, untreated and genistein-treated LM8 cells were subcutaneously inoculated into the backs of nude mice, and whether they developed metastatic tumors in the lung was histochemically examined. The main purpose of this study is to investigate the association of the expression of cytoplasmic β-catenin in primary tumor cells with metastatic potential. Therefore, the expression of β-catenin within the primary tumor was immunohistochemically examined. In addition, whether the metastatic potential of primary tumor cells is associated with the expression of MMP-2 was also examined.

## Results

### The expression of β-catenin in untreated and genistein-treated LM8 cells

**Figure 1:**
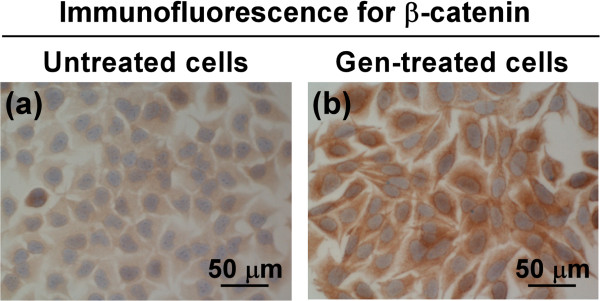
**The expression of β-catenin in untreated and genistein-treated LM8 cells.** LM8 cells were treated for 3 days without **(a)** or with **(b)** 50 μM genistein, and immunohistochemical staining of β-catenin was performed. Gen-treated cells are genistein-treated LM8 cells. Magnification: ×40.

LM8 cells were treated for 3 days without or with 50 μM genistein and fixed with ethanol. The expression of β-catenin in untreated and genistein-treated LM8 cells was immunohistochemically examined. In untreated LM8 cells, positive β-catenin immunostaining was observed in the cytoplasm and/or nucleus, and the intensity of immunostaining in the cytoplasm was weak (Figure 1a). In genistein-treated LM8 cells, positive β-catenin immunostaining was predominantly observed in the cytoplasm, and the intensity of immunostaining was stronger than that observed in untreated LM8 cells (Figure 1b). These findings indicate that genistein-treated LM8 cells expressed higher levels of cytoplasmic β-catenin than untreated LM8 cells.

### Growth and metastasis of untreated and genistein-treated LM8 cells in nude mice and C3H mice

**Figure 2:**
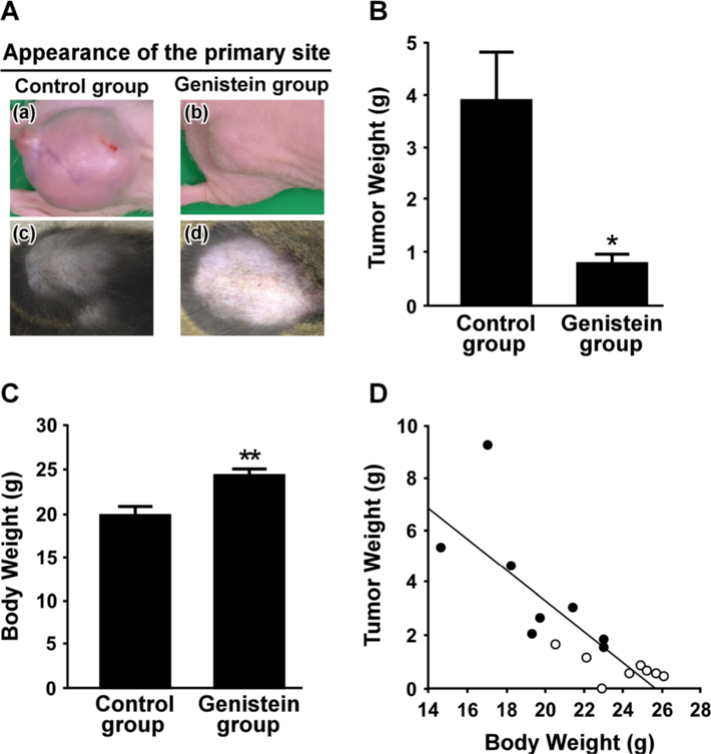
**Growth of LM8 cells in the control and genistein groups. (A)** Appearance of the inoculation site of the control **(a, c)** and genistein **(b, d)** groups. **(a, b)** nude mice at 25 days after tumor inoculation. **(c, d)** C3H mice at 36 days after tumor inoculation. **(B)** At 25 days after tumor inoculation into the backs of nude mice, tumor weight was measured. Values given are the means ± SE for eight mice in the control group and for seven mice in the genistein group. *p < 0.05 (compared with the control group) **(C)** At 25 days after tumor inoculation into the backs of nude mice, body weight was measured after the excision of the tumor. Values given are the means ± SE for eight mice in both groups. **p < 0.01 (compared with the control group) **(D)** Correlation between body weight and tumor weight in nude mice (n = 16) is shown. (●) the control group; (○) the genistein group.

Untreated and genistein-treated LM8 cells were harvested by trypsinization, centrifuged, resuspended in genistein-free culture medium, and inoculated subcutaneously into the backs of nude mice. Mice inoculated with untreated LM8 cells were termed the control group and those inoculated with genistein-treated LM8 cells were termed the genistein group. In the control group, all mice exhibited large tumors measuring 1.6-3.0 cm at the inoculation site (Figure 2A-a). The engraftment rate of tumor cells, which was calculated by dividing the number of tumor-bearing mice by the total number of mice, was 100% (Table 1). In the genistein group, one mouse did not exhibit tumors at the inoculation site and the remaining seven mice exhibited smaller tumors measuring 0.6-1.6 cm compared with the control group (Figure 2A-b). The engraftment rate of tumor cells was 87.5% (Table 1). The tumor weight was 3.85 ± 0.91 g in the control group and 0.89 ± 0.16 g in the genistein group (p < 0.05) (Figure 2B), indicating that genistein-treated LM8 cells grew at lower growth rate compared with untreated LM8 cells. The body weight was 19.5 ± 1.0 g in the control group (n = 8), and 24.0 ± 0.7 g in the genistein group (n = 8; p < 0.01) (Figure 2C). The body weight correlated negatively with the tumor weight [*r* = -0.812 (n = 16), p < 0.0001] (Figure 2D). Thus, the body weight decreased with the growth of the primary tumor.

**Table 1:** Engraftment rate and metastatic incidence of the control and genistein groups

	**Engraftment rate**	**Metastatic incidence**	
		**Lung**	**Liver**
Nude mice			
Control group	8/8 (100%)	8/8 (100%)*	7/8 (87.5%)*
Genistein group	7/8 (87.5%)	0/7 (0%)*	1/7 (14.3%)*
C3H mice			
Control group	7/7 (100%)	7/7 (100%)^§^	4/7 (57.1%)^§^
Genistein group	0/7 (0%)	0/7 (0%)^§^	0/7 (0%)^§^

*The sections of formalin-fixed, paraffin-embedded lungs and livers were stained with H&E, and metastatic tumors were microscopically confirmed.

^§^Multiple metastatic nodules at the surface of lungs and livers were macroscopically confirmed using a magnifying glass.

**Figure 3:**
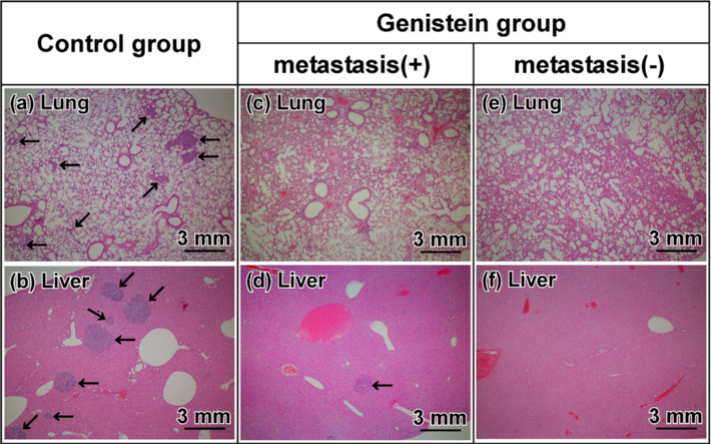
**Existence of metastatic tumors in the lung and liver.** The sections of formalin-fixed, paraffin-embedded lung **(a, c, e)** and liver **(b, d, f)** were stained with H&E. Arrows: metastatic tumor. **(a, b)** the control group. **(c-f)** the genistein group. Magnification: ×4.

To examine the presence of metastatic tumors in nude mice, the sections of formalin-fixed, paraffin-embedded lungs and livers were stained with H&E and observed microscopically under low magnification (×4). In the control group, multiple metastatic tumors were found in the lung (Figure 3a) and liver (Figure 3b) and the metastatic incidence was 100% in the lung and 87.5% in the liver (Table 1). In the genistein group, one exhibited the presence of the metastatic tumor in the liver (Figure 3d), but not the lung (Figure 3c). The remaining six mice did not exhibit the presence of any metastatic tumors in the lung (Figure 3e) or liver (Figure 3f), and this group was termed the genistein/metastasis(-) subgroup. The metastatic incidence in the genistein group was 0% in the lung and 14.3% in the liver (Table 1).

In another series of experiments, untreated and genistein-treated LM8 cells were subcutaneously inoculated into the backs of C3H mice. In the control group (n = 7), all mice exhibited large tumors measuring 0.7-1.7 cm at the inoculation site (Figure 2A-c). The engraftment rate of tumor cells was 100% (Table 1). The tumor weight of this group was 1.17 ± 0.20 g. Multiple metastatic nodules were macroscopically identified at the surface of the lung and liver (data not shown), and the metastatic incidence was 100% in the lung and 57.1% in the liver (Table 1). In the genistein group (n = 7), no mice exhibited any tumors at the inoculation site (Figure 2A-d) and developed metastatic nodules at the surface of the lung and liver (data not shown). Both the engraftment rate of tumor cells and metastatic incidence were 0% (Table 1).

### Expression of β-catenin within the primary and metastatic tumors in nude mice

**Figure 4:**
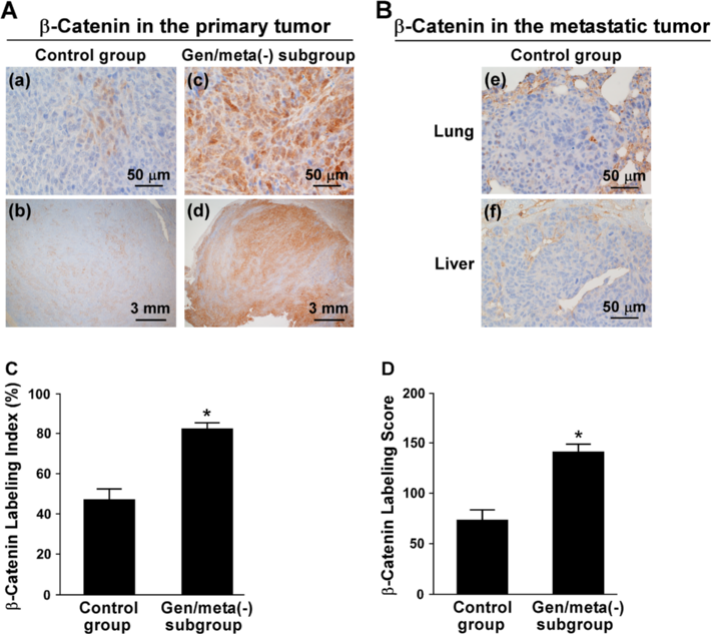
**Expression of β-catenin in the primary and metastatic tumors. (A)** Immunohistochemistry for β-catenin was performed using the primary tumor sections. **(a, b)** the control group. **(c, d)** Gen/meta(-) subgroup is the genistein/metastasis(-) subgroup. Magnification: **(a, c)** × 40; **(b, d)** × 4. **(B)** Immunohistochemistry for β-catenin was performed using the sections of the lung **(e)** and liver **(f)** in the control group. Magnification: ×40. **(C and D)** The β-catenin-labeling index **(C)** and β-catenin-labeling score **(D)** were determined. Gen/meta(-) subgroup is the genistein/metastasis(-) subgroup. Values given are the means ± SE for eight tumor specimens in the control group and for six tumor specimens in the genistein/metastasis(-) subgroup.

The expression of β-catenin within the primary tumors was immunohistochemically examined. Positive β-catenin immunostaining was predominantly observed in the cytoplasm of tumor cells (Figure 4A-a and 4A-c). In the control group, β-catenin-positive cells were sparsely observed within the primary tumor (Figure 4A-b), and the β-catenin-labeling index was 47 ± 6% (n = 8) (Figure 4C). Since the intensity of immunostaining varied significantly, the β-catenin-labeling score was also evaluated. The β-catenin-labeling score in the control group was 73 ± 10 (n = 8) (Figure 4D). In the genistein/metastasis(-) subgroup, β-catenin-positive cells were extensively observed within the primary tumor (Figure 4A-d), and the intensity of immunostaining was stronger compared with the control group (Figure 4A-c). The labeling index [82 ± 3% (n = 6)] and labeling score [140 ± 8 (n = 6)] for β-catenin were higher (p < 0.01) than those of the control group (Figure 4C and 4D).

The metastatic tumors in the lung (Figure 4B-e) and liver (Figure 4B-f) also expressed β-catenin in the cytoplasm, but the intensity of immunostaining was weak although endothelial cells of the blood vessels in the tumor were strongly immunostained.

### Expression of MMP-2 within the primary tumor in nude mice

**Figure 5:**
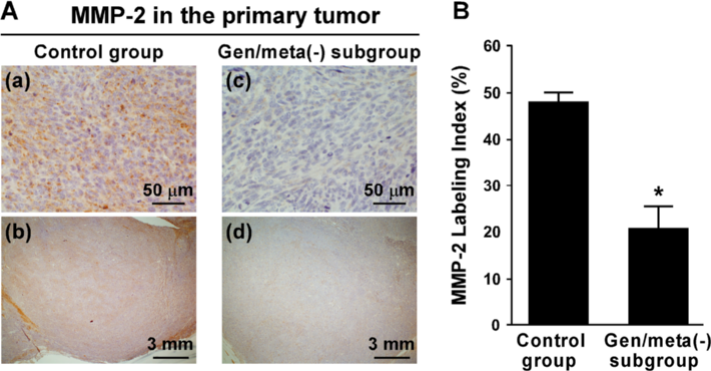
**Expression of MMP-2 in the primary tumors. (A)** Immunohistochemistry for MMP-2 was performed using the primary tumor sections. **(a, b)** the control group. **(c, d)** Gen/meta(-) subgroup is the genistein/metastasis(-) subgroup. Magnification: **(a, c)** × 40; **(b, d)** × 4. **(B)** The MMP-2-labeling index was determined. Gen/meta(-) subgroup is the genistein/metastasis(-) subgroup. Values given are the means ± SE for eight tumor specimens in the control group and for six tumor specimens in the genistein/metastasis(-) subgroup.

The expression of MMP-2 within the primary tumor was immunohistochemically examined. Positive MMP-2 immunostaining was observed in the cytoplasm of tumor cells (Figure 5A-a and 5A-c). In the control group, MMP-2-positive cells were extensively observed within the primary tumor (Figure 5A-b), and the MMP-2-labeling index was 48 ± 2% (n = 8) (Figure 5B). In the genistein/metastasis(-) subgroup, the primary tumor contained fewer MMP-2-positive cells compared with the control group (Figure 5A-d), and the MMP-2-labeling index [20 ± 5% (n = 6)] was lower (p < 0.01) than that of the control group (Figure 5B).

## Discussion

The purpose of this study was to investigate *in vivo* whether the level of cytoplasmic β-catenin in LM8 cells affected metastatic potential. To this end, we first examined whether untreated and genistein-treated LM8 cells metastasized to the distant organs in nude mice because genistein-treated LM8 cells expressed higher levels of cytoplasmic β-catenin than untreated LM8 cells (Figure 1). In the control group, primary tumor cells formed metastatic lesions in the lung and/or liver of all nude mice (Table 1). This is compatible with the previous reports stating that LM8 cells show an extremely high incidence of pulmonary metastasis in mice [9-12]. In the genistein group, primary tumor cells did not form metastatic lesions in the lung of all nude mice and the liver of 85.7% of nude mice (Table 1). This finding indicates that a majority of primary tumor cells in the genistein group lost metastatic potential.

Next, we performed immunohistochemical staining of β-catenin within the primary tumor. In the control group, 53% of tumor cells within the primary tumor were β-catenin-negative (Figure 4C), and the remaining 47% were β-catenin-positive but the intensity of immunostaining was weak or intermediate (Figure 4A-a). In the genistein/metastasis(-) subgroup, 82% of tumor cells within the primary tumor were β-catenin-positive (Figure 4C) and the intensity of immunostaining was stronger compared with the control group (Figure 4A-c). The results of β-catenin-labeling score showed that primary tumor cells in the genistein/metastasis(-) subgroup contained 1.9-times higher level of cytoplasmic β-catenin than those in the control group (Figure 4D). Based on these findings, we concluded that overexpression of cytoplasmic β-catenin in LM8 cells caused loss of metastatic potential to the lung and liver. Kashima et al. introduced *N-cadherin* and *cadherin-11* cDNAs into LM8 cells, in which there was little endogenous expression of these two cadherins, to investigate the role of the cadherins in osteosarcoma metastasis *in vivo*[20]. They found that the primary tumor of C3H mice injected with cadherin-transfected LM8 cells contained higher levels of cadherins compared with those injected with control, empty vector-transfected LM8 cells and that a high number of metastatic lesions were present in the lung of the latter mice, whereas there was a marked reduction in pulmonary metastases in the former mice. Based on these findings, they concluded that overexpression of cadherins attenuated the ability of LM8 cells to form pulmonary metastases.

Asai et al. [9] reported that subcutaneous inoculation of LM8 cells into the backs of C3H mice caused the rapid growth of tumor cells at the inoculation site and the formation of multiple metastatic nodules at the surface of the lung, and both the engraftment rate of tumor cells and metastatic incidence were 100%. The present study confirms this (Table 1). However, genistein-treated LM8 cells inoculated into the backs of C3H mice did not grow at the inoculation site and did not form metastatic nodules at the surface of the lung and liver (Table 1). Even in nude mice, the engraftment rate of the genistein group did not reach 100% (Table 1). Moreover, the metastatic incidence of this group was only 14.3%. These findings indicate that the malignancy of genistein-treated LM8 cells may be low. Since a majority of primary tumor cells in the genistein group was β-catenin-positive (Figure 4C), the present findings suggest that high expression of β-catenin within the primary tumor is associated with low malignancy of tumor cells. In human endometrial carcinoma, positive β-catenin expression has been reported to be associated with decreases in the stage and grade of the tumor [21,22]. Athanassiadou et al. [21] reported that loss of β-catenin is a strong and independent predictor of an unfavorable outcome in patients with endometrial carcinoma. In human gastric cancer, decreased expression of E-cadherin and catenins, including β-catenin, correlated with poor differentiation [23,24].

Invasion of tumor cells into the basement membrane is a critical event for tumor metastasis. Invasive tumors exhibit high levels of MMPs [8,9,12,17]. MMPs are capable of digesting various components of the extracellular matrix (ECM) and play a pivotal role in tumor metastasis by removing physical barriers to invasion [18,19]. In particular, MMP-2 degrades ECM macromolecules in the basement membranes and other interstitial connective tissues [18]. Asai et al. [9] reported that LM8 cells secreted higher levels of MMP-2 and exhibited extremely higher invasiveness *in vitro* compared with Dunn murine osteosarcoma cells with no metastatic potential to the lung. Our previous *in vitro* study showed that genistein-treated LM8 cells secreted lower levels of MMP-2 and were less invasive compared with untreated LM8 cells [8]. Moreover, our previous study with nude mice inoculated with LM8 cells showed that decreased expression of MMP-2 within the primary tumor was associated with the suppression of the development of metastasis in the lung [12]. Our present study showed that a majority of primary tumor cells of the genistein/metastasis(-) subgroup was MMP-2-negative (Figure 5A-c). The percentage of MMP-2-negative cells to total cells in this subgroup was 80 ± 5% (Figure 5B). This value was similar to that of the β-catenin-labeling index (82 ± 3%) in this subgroup. Taken together, our present findings suggest that decreased expression of MMP-2 in β-catenin-overexpressing LM8 cells may cause the prevention of local invasion, thus resulting in inhibition of the growth of primary tumor and the metastasis to the lung and liver.

In this study, we performed heat-induced antigen retrieval in 10 mM citrate buffer (pH 6.0) for immunohistochemical staining of β-catenin and showed that the primary tumor in the control group expressed lower level of cytoplasmic β-catenin compared with the genistein/metastasis(-) subgroup (Figure 4A). Moreover, we found that the metastatic tumor in the lung and liver also expressed very low level of cytoplasmic β-catenin (Figure 4B). Kashima et al. [25] also performed antigen retrieval in citrate acid buffer and showed low expression of cytoplasmic β-catenin in human primary osteosarcoma with metastasis and human metastatic osteosarcoma. Thus, osteosarcoma with metastatic potential seems to exhibit low expression of cytoplasmic β-catenin when heat-induced antigen retrieval was performed under acidic pH. Iwaya et al. [10] performed heat-induced antigen retrieval in 10 mM citrate buffer (pH 8.0) and showed that the expression of cytoplasmic and/or nuclear β-catenin within the primary tumor was higher in C3H mice inoculated with LM8 cells than in those inoculated with Dunn cells. Moreover, they found that in human metastatic osteosarcoma, more than 10% of tumor cells were immunostained for β-catenin in the cytoplasm and/or nucleus [10]. These findings are inconsistent with ours. This inconsistency may be due to the different pH utilized in heat-induced antigen retrieval because the efficiency of heat-induced antigen retrieval is dependent on the pH of the retrieval solutions [26-28].

Preclinical and clinical studies have shown that protein kinases, which are involved in the regulation of a wide variety of cellular processes, are relevant targets for cancer therapy [29,30]. Bruzzese et al. [31] reported that treatment of Hep-2 cells with gefitinib, a tyrosine kinase inhibitor [29], inhibited tyrosine phosphorylation of epidermal growth factor receptor and decreased invasive potential. Genistein also is a specific and potent inhibitor of tyrosine kinase [32,33]. We previously found that genistein decreased motile and invasive potential of LM8 cells [8]. Whether genistein inhibited tyrosine phosphorylation of proteins in LM8 cells remains unclear. It is unlikely, however, that high expression of cytoplasmic β-catenin in genistein-treated LM8 cells results from inhibition of tyrosine phosphorylation of β-catenin by genistein because phosphorylation of β-catenin by tyrosine kinase leads to an increase in the free pool of cytoplasmic β-catenin during epithelial cell migration [34]. This interpretation may be also supported by reports stating that tyrosine phosphorylation of cell-cell adhesion molecules, including β-catenin, affected their functions, causing unstable cell-cell adhesion and migration of cells [35-37].

## Conclusions

Overexpression of cytoplasmic β-catenin in LM8 cells causes inhibition of the growth of primary tumors and loss of metastatic potential to the lung and liver. Therefore, overexpression of cytoplasmic β-catenin within the primary osteosarcoma may indicate the absence of metastatic lesions at distant organs when heat-induced antigen retrieval for immunohistochemical staining was performed under acidic pH.

## Methods

### Animals, cells, reagents, and antibodies

Male BALB/cA Jcl-*nu* nude mice (4-week-old) and male C3H mice (4-week-old) were obtained from CLEA Japan, Inc., Tokyo, Japan. LM8 cells (RBRC-RCB1450) were obtained from RIKEN BRC Cell Bank, Ibaraki, Japan. Genistein (Sigma-Aldrich, St. Louis, MO) was dissolved in DMSO. For immunohistochemical staining, a rabbit polyclonal antibody to β-catenin (sc-7199; Santa Cruz Biotechnology, Santa Cruz, CA) and a mouse monoclonal antibody to MMP-2 (NCL-MMP2-507; Novocastra Reagent, Leica Microsystems, North Ryde, Australia) were diluted to 1:100 and 1:80, respectively, with phosphate-buffered saline (PBS).

### Cell culture

LM8 cells (1.25 × 10^3^ cells/cm^2^) were seeded on a 60 mm plate in culture medium, which contained 10% fetal bovine serum, 100 units/ml penicillin, and 100 μg/ml streptomycin in Dulbecco’s modified Eagle’s medium. After 24 h of seeding, the medium was replaced with culture medium with or without 50 μM genistein. Cells were incubated for 3 days, harvested by trypsinization, centrifuged at 1,000 × *g* for 10 min, and resuspended in genistein-free culture medium for inoculation.

### Tumor inoculation

The suspensions (1 × 10^5^ cells/0.3 ml of culture medium) of untreated and genistein-treated cells were subcutaneously inoculated into the backs of nude mice and C3H mice under ether anesthesia. Two mice were housed in a standard polypropylene mouse cage in a 12 h light–dark cycle (lights on at 7 am and off at 7 pm) and were allowed free access to laboratory chow and water. After 25 (for nude mice) and 36 (for C3H mice) days of inoculation, the animals were sacrificed under ether anesthesia. In nude mice, the tumors, lungs, and livers were excised, weighed, fixed in 10% formalin, and embedded in paraffin. The sections (4 μm) of formalin-fixed, paraffin-embedded lungs and livers were deparaffinized, rehydrated, and stained with H&E to confirm microscopically the absence or presence of metastatic tumors. In C3H mice, the tumors were excised and weighed. The lungs and livers were excised and observed macroscopically using a magnifying glass to confirm the absence or presence of metastatic nodules at the surface.

All animals were treated humanely, and care was taken to alleviate suffering. The experimental protocols were reviewed and approved by the local Animal Ethics Committees at the Ehime University Graduate School of Medicine, Ehime, Japan.

### Immunohistochemical studies

The sections (4 μm) of formalin-fixed, paraffin-embedded tumors, lungs, and livers were deparaffinized and rehydrated, which were followed by heat-induced (30 min at 98°C) antigen retrieval in 10 mM citrate buffer (pH 6.0) for β-catenin, and in 1 mM EDTA solution (pH 8.0) for MMP-2. The sections were incubated for 1 h with a primary antibody and were then incubated for 1 h with EnVision™+ DualLink (Dako Japan, Inc., Tokyo, Japan), as described previously [11]. Positive cells were visualized by adding 3,3′-diaminobenzidine tetrahydrochloride (DAB; Dako Japan) to the sections. The nuclei were counterstained with hematoxylin.

To determine the labeling index for β-catenin and MMP-2 and the labeling score for β-catenin, the tumor sections were observed microscopically under high-power magnification (×40), and three different microscopic fields per section were photographed. Then, β-catenin-positive or MMP-2-positive cells present in approximately 500 cells per photograph were counted. The labeling index was evaluated by determining the percentage of the number of positive cells to the total number of cells. To determine the labeling score, β-catenin expression was estimated “0” if negative, “1+” if week intensity, and “2+” for intermediate or strong intensity, as described previously [38]. The β-catenin-labeling score was evaluated as follows: β-catenin-labeling score = [(1 × number of “1+” cells + 2 × number of “2+” cells)/total number of cells] × 100. The total number of cells is the sum of numbers of “0”, “1+”, and “2+” cells. Values for three fields per tumor section were averaged to obtain the labeling index and labeling score for each tumor.

In another series of experiments, LM8 cells (1.19 × 10^3^ cells/cm^2^) were incubated for 24 h on a 2-well chamber slide (Nalge Nunc International, Osaka, Japan). Then, cells were treated for 3 days without or with 50 μM genistein, fixed in 70% ethanol for 30 min, incubated in 100% ethanol for 10 min, washed twice with PBS, and incubated for 1 h with a rabbit polyclonal antibody to β-catenin (1:15 dilution in PBS containing 1% bovine serum albumin) followed by 1-h incubation with EnVision™+ DualLink. Positive cells were visualized by adding DAB. The nuclei were counterstained with hematoxylin. Cells were then mounted in glycergel (Dako Japan) for light microscopy analysis (magnification: ×40).

### Statistical analyses

Significant differences between two independent groups were analyzed using Student’s *t*-test. Pearson’s *r* was used to calculate the correlation between the body weight and the tumor weight. For all statistical analyses, the criterion for significance was p < 0.05. All values were expressed as the means ± SE.

## Competing interests

The authors declare that they have no competing interests.

## Authors’ contributions

TK and AN performed the bulk of experiments in vitro and in vivo and contributed equally to this study. SK participated in experiments including tumor inoculation and tumor removal. YN participated in immunohistochemical study. HM (H. Miura) designed experiments and analyzed data. HM (H. Masuno) is a project leader, designed experiments, analyzed data, and wrote the manuscript. All authors read and approved the final manuscript.
